# Biological variation of human aggrecan ARGS neoepitope in synovial fluid and serum in early-stage knee osteoarthritis and after knee injury

**DOI:** 10.1016/j.ocarto.2022.100307

**Published:** 2022-08-27

**Authors:** Staffan Larsson, L. Stefan Lohmander, André Struglics

**Affiliations:** Lund University, Faculty of Medicine, Department of Clinical Sciences Lund, Orthopaedics, Lund, Sweden

**Keywords:** Aggrecan, Aggrecanase, ARGS, Biomarkers, Biological variation

## Abstract

**Objective:**

To determine biological variation of the aggrecanase-generated aggrecan ARGS neoepitope in serum (sARGS) and synovial fluid (sfARGS) within and between patients with osteoarthritis (OA) or anterior cruciate ligament (ACL) injury.

**Design:**

Matched samples of serum and synovial fluid were available, as parts of clinical trials, from i) 16 subjects with early-stage OA on 8 occasions over 1 year, and ii) 120 subjects with acute ACL injury with samples available from at least 2 of 6 visits over 5 years. We used an in-house immunoassay to quantify ARGS and one-way ANOVA for statistical analyses.

**Results:**

Variability in ARGS was higher in synovial fluid than in serum in both patient groups. Subjects with OA had the lowest variability both within and between patients and showed no variation over time in the degree of variability or in the cross-sectional mean, neither in serum nor in synovial fluid. After ACL injury, the concentration and the variability of ARGS was highest immediately after injury, with a subsequent decline both in concentration and in variability with time. In both patient groups there was a positive correlation between sfARGS and sARGS both within and between individuals (correlation coefficients between 0.16 and 0.20).

**Conclusions:**

The biological variation of ARGS is lower in serum than in synovial fluid, and lower in OA than after ACL injury. Serum ARGS is a measure of the total release of ARGS aggrecan from the whole body and a poor reflection of the release of ARGS aggrecan within the affected joint.

## 1 Introduction

There is a great need for biomarkers of osteoarthritis (OA) [1]. The field of application is wide and ranges from patient diagnostics, prognosis and personalized management of OA to monitoring molecular effects and mechanisms in drug development [1,2]. In the ongoing, not yet successful, search for a disease-modifying osteoarthritis drug (DMOAD), one recent focus involves inhibition of the aggrecanase ADAMTS-5 (a disintegrin and metalloproteinase with thrombospondin motifs), identified as a key protease in pathological degradation of human aggrecan [[3], [4], [5]]. To monitor aggrecanase activity, and inhibition thereof, quantification of the aggrecanase generated aggrecan amino acids alanine, arginine, glycine, serine (ARGS) neoepitope in synovial fluid or serum has been used [[6], [7], [8]]. To be able to plan, power and interpret such studies, we need to know the biological variation of the biomarker, both between patients and within patients over time. Such information is generally lacking for OA biomarkers. Only one prior study reported on measures of longitudinal and cross-sectional variability in markers of extracellular matrix metabolism [9], while some studies inform on diurnal variation or variation related to exercise [[10], [11], [12], [13]].

For aggrecanase-generated ARGS several studies have reported on differences between patient groups [11,[14], [15], [16]], temporal changes after rupture of the anterior cruciate ligament (ACL) of the knee [[17], [18], [19], [20]], and on diurnal variation in serum [11,12]. None of these studies have reported on measures of variability. We here describe the biological variation of the aggrecan ARGS neoepitope within individuals over time and between individuals in both knee joint fluid and serum in two target populations for DMOADs: early-stage knee OA and ACL injury.

## 2 Patients and methods

### 2.1 Subjects and samples

Subjects were selected from two previous study cohorts.

#### 2.1.1 Early-stage OA

We analyzed samples available from the ARTZAL-study comprising 52 patients with knee pain and arthroscopically verified deep cartilage fissures and villous-like flakes in the symptomatic knee, considered to represent early-stage OA, randomly assigned to receive intraarticular injections of 2.5 ​ml of either hyaluronan or vehicle, weekly for 5 weeks, and followed-up for one year [21]. No difference was noted between those assigned to hyaluronan or placebo treatment in any of the primary outcomes (subjective rating of total knee function, pain in the knee, range of motion, and activity level, scored using 100 ​mm visual analog scale, and knee function scored using the Lysholm scale [22]) or secondary outcomes [21], nor in the concentration of ARGS in synovial fluid and serum (Supplemental Figure S1). Knee-puncture and blood sampling was done on eight time points (baseline and at weeks 1, 2, 3, 4, 13, 26, and 52). A complete set of matched samples of synovial fluid and serum were available from 16 subjects, which were included in the present study. Mean age of the included subjects was at baseline 45 years (range 31–59), 4 were females, 5 had a ruptured anterior cruciate ligament more than 1 year before inclusion, and 7 received hyaluronan and 9 placebo.

#### 2.1.2 ACL injury

From the KANON trial (ISRCTN 84752559) [23] comprising 121 subjects, we used previously published data on synovial fluid and serum ARGS on 120 subjects (27% women, mean age 26 years, SD 4.9, Supplemental Table S1) with an acute ACL rupture to a previously un-injured knee, which were followed at baseline (0–6 weeks after injury), 2 and 5 years after injury, with additional follow ups at 4, 8 and 12 months for the 63 first included subjects [17,19]. In addition to a similar structured rehabilitation protocol administered to all subjects, 62 were randomized to undergo early ACL reconstruction performed within 10 weeks after injury, and 59 to the option of having a delayed ACL reconstruction if needed [23]. No difference was observed between treatments for the primary outcome measure change in the average score on four subscales of the knee injury and osteoarthritis outcome score (KOOS_4_) from baseline to 2 or 5 years [23,24], or in the temporal change after injury in synovial fluid and serum ARGS [19]. We excluded one subject which lacked longitudinal biomarker data (samples taken at baseline only). All available data on ARGS on 120 subjects was included in this study (545 data points for serum ARGS and 347 for synovial fluid ARGS, Supplemental Table S1), although not always used in statistical calculation (e.g., when matched samples were required) or in graphical presentations (where any selection is explained in the figure legends).

### 2.2 Ethics

Approval was given by the regional ethical review board in Lund (Number LU361-00 for the ARTZAL study, and LU535-01 for the KANON study). All participants gave written informed consent to participate.

### 2.3 Methods

For assessment of aggrecanase generated ARGS in synovial fluid and serum we used an in-house immunoassay on the Mesoscale Discovery (MSD) platform, with the anti-aggrecan AHP0022 (Invitrogen) as a capture antibody and the neo-specific monoclonal OA-1 antibody against the ARGS neoepitope for detection, using ADAMTS-4 digested human recombinant G1-G2 peptide (R&D, 1220-PG-25) as standard [25]. To increase the sensitivity of the assay, samples are deglycosylated prior to analysis using Chondroitinase ABC and Keratanase as described [25]. The coefficients of variation (CV) within and between plates for this assay were established in the KANON cohort as 2.9% and 22.7% for serum and 4.2% and 13.7% for synovial fluid [25]. In the analysis of the early-stage OA cohort the specific between-plate CV of the quality control samples were 3.4% for synovial fluid on 6 plates and 11.6% for serum on 4 plates. In this study we analyzed all samples from the same individual of the same fluid type on the same plate, and all samples were within the detection limits of the assay.

For clarity, when needed the fluid of origin of the biomarkers is indicated by the addition of a prefix to the biomarker: synovial fluid (sf) and serum (s).

### 2.4 Statistics

We used a one-way ANOVA to calculate the different measures of variance for ARGS in synovial fluid and serum: standard deviation (SD) and coefficient of variation (CV) between and within individuals, and intraclass correlation one (ICC_1_). The ICC_1_ is the ratio between the variance between individuals and the sum of the variance within and between individuals and can be interpreted as the total amount of variance that is attributable to between individual rather than within individual difference over time. Calculations of within subject and between subject correlation of ARGS in synovial fluid and serum was done according to Bland and Altman [26,27]. In the above calculations, we excluded the baseline samples from the ACL injury subjects of the KANON study, since these synovial fluid and serum samples were obtained at different time points after injury – synovial fluid at the acute visit and serum at the randomization visit on average 11 days (range 1–24 days) later. For cross-sectional correlation at each visit (Fig. 4B) we used Person's correlation on all available data. To compare mean ARGS-concentrations between visits in the early-stage OA cohort we used an ANOVA with repeated measures with a Greenhouse-Geisser correction.

## 3 Results

**Fig. 1:**
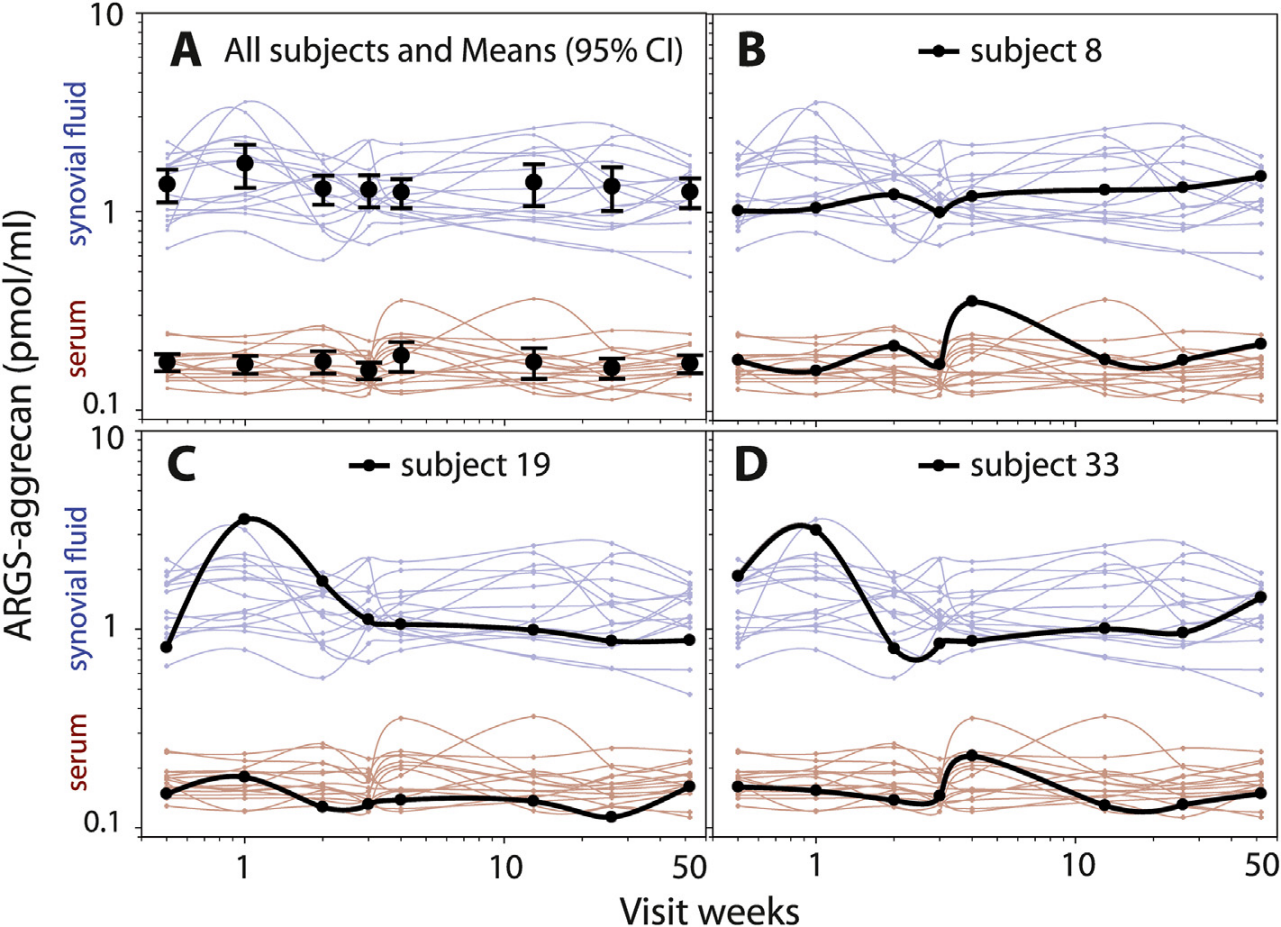
**Temporal variation of ARGS in synovial fluid and serum in 16 subjects with early-stage OA over 1 year in synovial fluid (blue) and serum (red). (A)** All subjects with means and 95% confidence intervals. **(B**–**D)** Examples of three individual subjects highlighted as bold black lines. All curves are drawn using spline interpolation.

In the early-stage OA cohort, the concentration of ARGS was on average 8 times higher in synovial fluid than in serum (Table 1). There were no statistically significant differences between visits in the mean concentrations of ARGS in synovial fluid and serum over the one-year period (Fig. 1A, Supplemental Table S2). The stability over time of ARGS concentrations in both synovial fluid and serum was further visible as no change over one year in the proportional difference compared to the overall mean of the same subject (Fig. 3). The individual variation over time is shown for all as line graphs (Fig. 1A).

**Table 1:** Biological variation of ARGS within and between individuals.

	Early-stage OA over 1 year		ACL injury 4 months to 5 years after injury		ACL injury Baseline, 0–44 days after injury	
Body fluid (n samples^a^)	sf (128)	s (128)	sf (300)	s (425)	sf (47)	s (120)
Overall mean, pmol/ml (range)	1.37 (0.47–3.58)	0.17 (0.11–0.36)	1.25 (0.19–6.38)	0.13 (0.03–0.36)	11.68 (0.56–50.82)	0.17 (0.06–0.80)
Within subject SD, pmol/ml	0.44	0.03	0.62	0.02	–	–
Between subjects SD, pmol/ml	0.33	0.03	0.38	0.04	11.48	0.09
Within subject CV	32%	16%	42%	13%	–	–
Between subjects CV	24%	18%	31%	34%	98%	52%
ICC^1^	0.354	0.525	0.278	0.841	–	–

a Details on number of samples per visit in Supplemental Table S1.

The biological variation of ARGS in these subjects with early-stage OA was higher in synovial fluid than in serum, both between and within individuals, as indicated by 1.3–2 times higher coefficient of variations in synovial fluid compared to serum (Table 1).

The ICC_1_ value for sARGS in the subjects with OA was 0.52, meaning that the total variation seen for sARGS derived in equal parts from between individual and within individual variation (Table 1). This was further indicated by the similarities in coefficients of variation within and between individuals in coefficients of variation (16% and 18%, respectively, Table 1). For sfARGS the ICC_1_ value was 0.35, indicating that the larger part of the total variation, approximately two-thirds, could be attributed to within-subject variation over time, and only one-third to between-subject variation in means (Table 1). This was further visible as a higher coefficient of variation within subjects compared to between subjects (32% and 24%, respectively, Table 1).

### 3.1 Correlation between sfARGS and sARGS in early-stage OA

In these 16 subjects with early OA, there was a positive correlation between ARGS in synovial fluid and serum, both between subjects (r ​= ​0.20) and within subjects (r ​= ​0.16) (Table 2). Although some correlation exists between ARGS in synovial fluid and serum within the group, examples of line graphs following single subjects over time show one subject which had a peak in sARGS at week four, without a corresponding peak in sfARGS (subject 8, Fig. 1B), and two subjects which had peaks in sfARGS at week one, without corresponding peaks in sARGS (subjects 19 and 33, Fig. 1C and D).

**Table 2:** Correlation between synovial fluid-ARGS and serum-ARGS within and between individuals.

	early-stage OA	ACL injury 4 months to 5 years after injury
Within-subject correlation	0.155	0.174
Between subjects correlation	0.205	0.165

Early -stage OA: 16 subjects at 5 time points. ACL injury: 120 subjects at 1 to 5 time points. Se Supplemental Table S1 for details on number of samples per visit.

### 3.2 Biological variation of ARGS after ACL injury

**Fig. 2:**
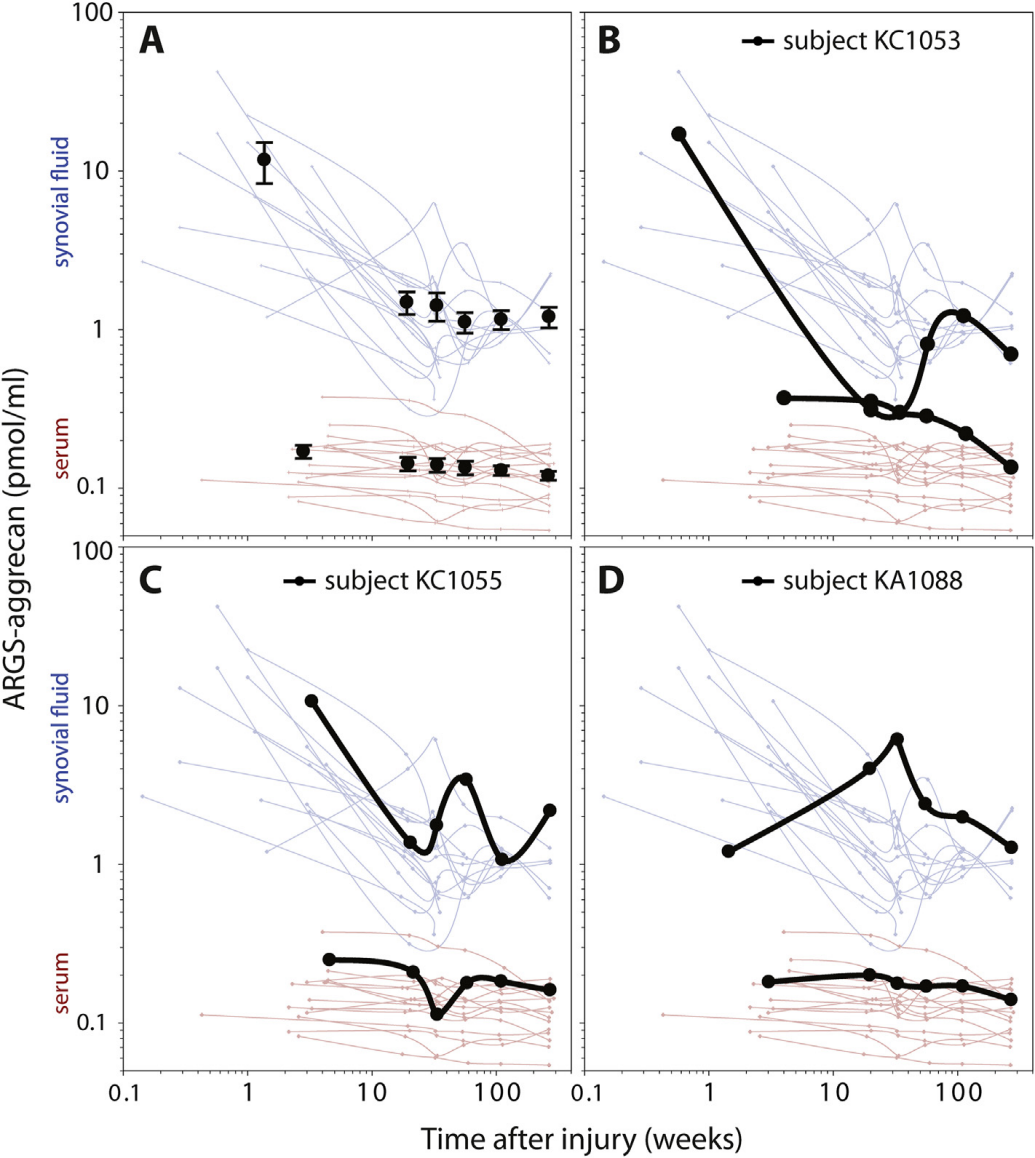
**Temporal variation of ARGS in synovial fluid and serum in subjects with ACL injury over 5 years in synovial fluid (blue) and serum (red). (A)** Circles and error bars show mean and 95% confidence intervals for all available data at each visit for the 121 subjects. Line graphs show longitudinal concentrations from the 16 individuals that had samples of synovial fluid and serum from baseline and at least three consecutive visits. **(B**–**D)** Examples of three individual subjects highlighted as bold black lines. All curves are drawn using spline interpolation.

**Fig. 3:**
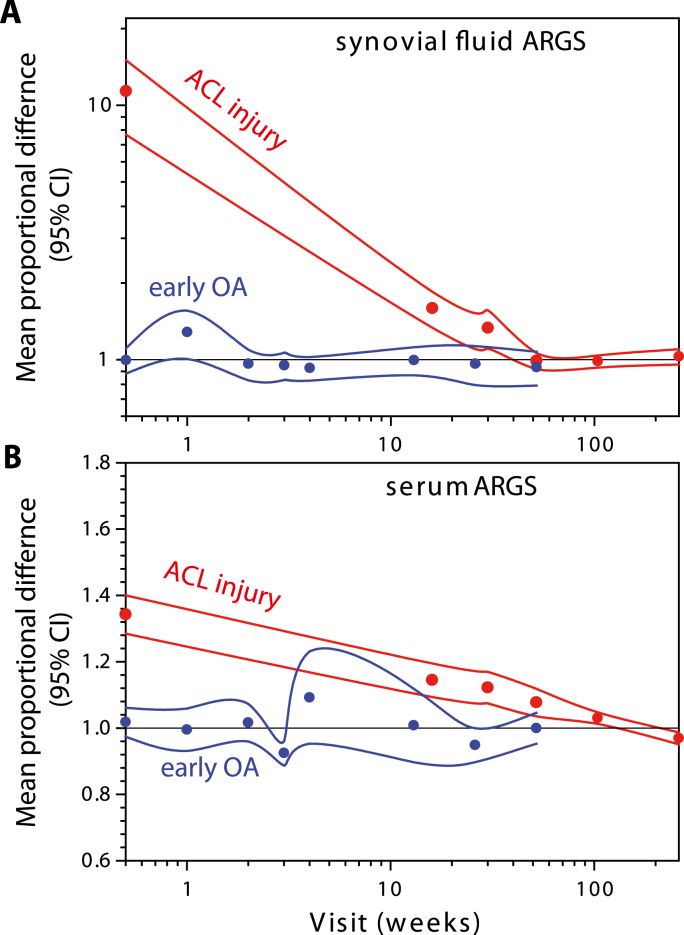
**Mean proportional difference in ARGS at each visit compared to stabilized mean concentration in subjects with early-stage OA (blue) and after ACL injury (red).** For each subject, difference was calculated as the ratio of the subjects ARGS at a visit over the mean ARGS in the subject after stabilization (for early-stage OA, mean of all visits; for ACL injury mean of the last two visits at 2 and 5 years). Circles, mean ratios at each visit with 95% confidence intervals of the means as solid lines drawn with spline fit. Black horizontal line, reference ratio of 1 indicating no difference versus mean or stabilized mean. Note that log scale was used for synovial fluid (A) and linear scale for serum (B) for optimal resolution and visibility. Curves are drawn using spline interpolation.

**Fig. 4:**
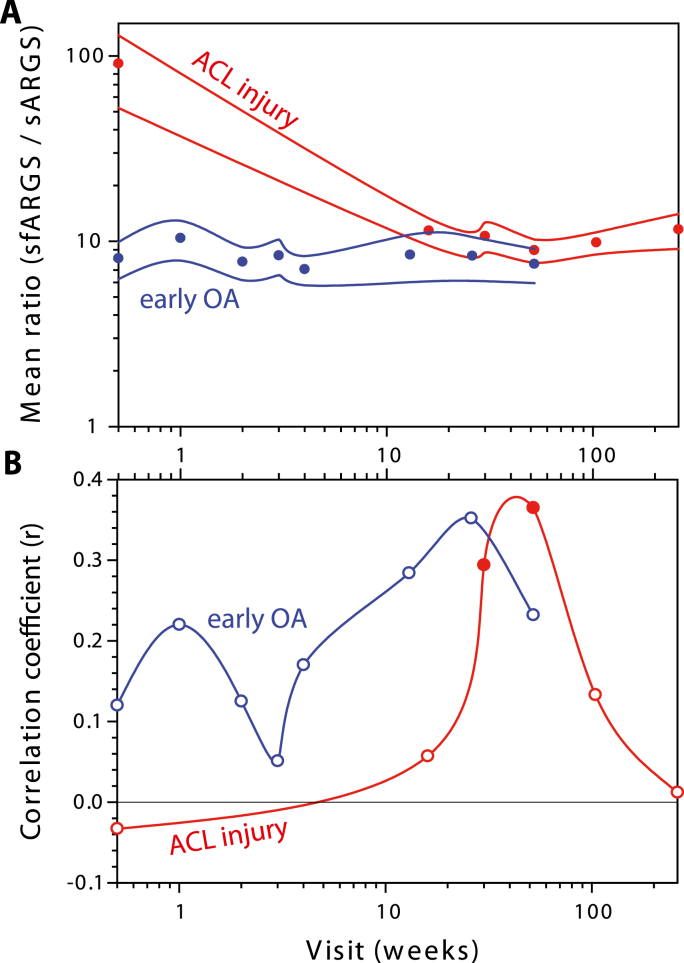
**Temporal relationship between ARGS in synovial fluid and serum in subjects with early-stage OA (blue) and with ACL injury (red). (A)** The ratio between synovial fluid ARGS and serum ARGS. Circles, mean ratios at each visit with 95% confidence intervals of the means as solid lines. **(B)** Pearson correlation between synovial fluid ARGS and serum ARGS at each visit (circles) with visits interconnected by solid lines. Filled circles indicate statistically significant correlation at an alpha level of 0.05. Black horizontal line, reference at 0 indicating no correlation. Curves are drawn using spline interpolation. Detailed data presented in Supplemental Table S3.

As reported, ARGS concentrations were elevated after ACL injury in both synovial fluid and serum with a subsequent temporal decrease towards normal concentrations (Fig. 2A) [17]. In the immediate days and weeks after an ACL injury, the proportional difference in ARGS concentration compared to stabilized mean concentrations at 2–5 years was 10-fold higher in synovial fluid and 1.4-fold higher in serum (Fig. 3). The difference in the magnitudes of elevation in synovial fluid and serum after injury was visible as a temporal relationship in the ratio between sfARGS and sARGS, where concentrations immediately after injury was 91 times higher in synovial fluid compared to in serum, with a subsequent decrease towards a mean ratio of 10 after 2–5 years (Fig. 4A).

The biological variation of ARGS in these subjects with ACL injury was highest at baseline and in synovial fluid where the between-subjects CV was 98%, 3 times higher than at follow up visits 4 months to 5 years after injury (CV ​= ​31%), and 4 times higher than in early-stage OA (CV ​= ​24%; Table 1). Although the variation at baseline in serum was approximately half of that seen in synovial fluid (between-subjects CV of 52%), it was still 1.5 times higher than at follow up visit 4 months to 5 years after injury (CV ​= ​34%), and 3 times higher than what was noted in early-stage OA (CV ​= ​18%; Table 1).

At the follow up visits 4 months to 5 years after ACL injury, the between-subjects CV was highly similar in synovial fluid and serum (31% and 34%, respectively), whereas the variation within subjects at 42% was more than 3 times higher in synovial fluid compared to the 13% in serum (Table 1).

The higher within-subject variation of ARGS in synovial fluid, was also visible as a low ICC_1_ value of 0.278 for synovial fluid, which indicates that approximately 70% of the total variation of sfARGS in synovial fluid could be attributed to within-subject variation (Table 1). In serum this was reversed, and the ICC_1_ of 0.841 indicated that more than 80% of the total variation of sARGS could be attributed to between-subject variation (Table 1).

### 3.3 Correlation between sfARGS and sARGS after ACL injury

In these 120 subjects with ACL injury, in the visits from 4 months to 5 years, there was a positive correlation between ARGS in synovial fluid and serum, both between subjects (r ​= ​0.17) and within subjects (r ​= ​0.16) (Table 2). However, examples of line graphs following individuals over time show one subject with an extreme dip in synovial fluid levels at visit weeks 16 and 30 (with a concentration in synovial fluid as low as in serum) without a corresponding dip in serum at the same visits (subject KC1053, Fig. 2B), and examples of subjects which display peaks in synovial fluid at follow up visits where the corresponding serum samples show either no peak or a dip in serum concentration at the same visit (subjects KC1055 and KA1088, Fig. 2C and D).

### 3.4 Temporal relationship between ARGS in synovial fluid and serum after ACL injury and in early-stage OA

The relationship between ARGS in synovial fluid and in serum, calculated as the ratio sfARGS to sARGS, changed with time after injury from an initial ratio of 91 acutely after injury to a ratio of approximately 10 in the following visits (Fig. 4A). In contrast, the early OA cohort had an over the entire one-year follow-up period stable ARGS-ratio of approximately 8.

The point estimates of the within-subject correlation measured at each visit spanned over a wider range and fluctuated more after ACL injury than in early OA (Fig. 4B).

## 4 Discussion

In two distinctly different patient groups – ACL injury and early-stage knee OA – both of which potentially could be treated by DMOADs involving inhibition of aggrecanases, we here report the biological variation of the aggrecanase generated ARGS neoepitope in joint fluid from the affected knee, as well as in serum. In addition to the reported measures of variability being important for planning, powering, and interpretation of studies, three main observations can be made. 1) The variation of ARGS is larger after injury than in early-stage OA. 2) The variation of ARGS is larger in synovial fluid than in serum. 3) Although ARGS in synovial fluid and serum are correlated, the individual variation is substantial, and within a patient a measurement in one fluid cannot substitute for measurement in the other.

Injurious compression of cartilage gives an immediate increased expression of the aggrecanase ADAMTS-5 without affecting the expression of aggrecan [28], suggestive of detrimental degradation, and not remodeling, of cartilage. The resulting increase in the release of the aggrecan ARGS neoepitope into synovial fluid and serum and the temporal changes after ACL injury have been reported in both synovial fluid and serum in the ACL injury cohort used here [17,19], with similar findings in serum in a different cohort of ACL-injured patients [20]. However, neither of those studies reported on the details of variability between and within individuals included in the present study.

In the ACL injury cohort, we chose to look at the variability in the acute phase after injury separately from the follow up period from 4 months to 5 years. The primary reason for this was that the baseline sampling of synovial fluid and serum was not done at the same time point, which complicates interpretation in differences in variability and correlation between synovial fluid and serum ARGS. The heterogeneity in baseline sampling (ranging from 0 to 44 days after injury), in combination with a known rapid change in both expression of aggrecanases after injury [28], and ARGS concentration in synovial fluid in the immediate time after injury [17], was a further reason to look at this visit and the follow-up visits separately. The variation in sampling time at baseline may explain some of the very high variability in ARGS between subjects at baseline, but the variability may also be due to differences in the magnitude of the initial joint trauma.

This is to our knowledge the first study that reports on variability of ARGS aggrecan in both serum and synovial fluid. The previous few studies have looked at short term variability in serum only. One study investigated the sensitivity of sARGS to exercise and found minute increases in the hours following moderate exercise, very low day-to-day variation in the individual, but a substantial variation between individuals [29]. Two publications showing line graphs of individual variation of sARGS over hours and a few weeks found no significant diurnal or inter-day variation in sARGS on the same 20 subjects with knee OA that was not end-stage [11,12]. In a cohort of subjects with well-defined early-stage OA, we here present similar line graphs for both serum and synovial fluid following subjects over one year, and novel data on measures of long-term variability both within and between subjects in serum as well as in synovial fluid, in both early-stage OA and after ACL injury.

We have previously noted that ARGS concentrations in serum and synovial fluid are correlated in subjects with ACL injury [17,25]. In this more detailed analysis, we corroborate those findings and in addition show that the concentration of ARGS aggrecan was correlated in synovial fluid and serum both between subjects and within subjects, in early-stage knee OA as well as after ACL injury. Correlation between subjects indicate that subjects with higher mean ARGS concentration in synovial fluid also tend to have higher mean concentration over time in serum. This may be interpreted as an overall and over time higher aggrecanase activity in an individual giving rise to greater ARGS release in a single joint as well as systemically. The within-subject correlation indicates that an increase of sfARGS in an individual on one occasion is associated with an increase also in sARGS at the same time. Such an association between ARGS in synovial fluid and serum within individuals may be useful at a group level in studies of many participants where synovial fluid is not accessible. However, at the individual level, line graphs following individuals over time show that high sARGS on one occasion was not always associated with high sfARGS at the same visit, and conversely, high sfARGS at one visit was not always reflected as an increase in sARGS at the same visit. We further note that, when studying the correlation between ARGS in synovial fluid and serum cross-sectionally, there is a considerable variation between visits, with point estimates of the correlation coefficients ranging from not statistically significant negative to positive 0.36. Despite some degree of correlation between sfARGS and sARGS, the combined data show that the ARGS concentration in one body fluid cannot be used as a proxy for concentration in the other. The reason for this may lay in that the systemic pool of ARGS is released from aggrecan from all synovial joints, not just the studied index joint. Although the main source of aggrecan is from synovial joints where articular cartilage, menisci, tendon and ligament fibrocartilage and synovium tissues express aggrecan [[30], [31], [32], [33]], other tissues, such an as intervertebral discs [34], the neural nets [35], and the vasculature [36], also express aggrecan and may contribute to the systemic pool of ARGS. Further degradation or processing of aggrecan fragments in the draining of synovial fluid through the lymphatic system may also contribute. We have noted that of the two dominating aggrecan ARGS species in synovial fluid – ARGS extending into the chondroitin sulphate rich region 1 (CS1) and ARGS-CS2 – mainly ARGS-CS1 is detected in serum [25].

**Fig. 5:**
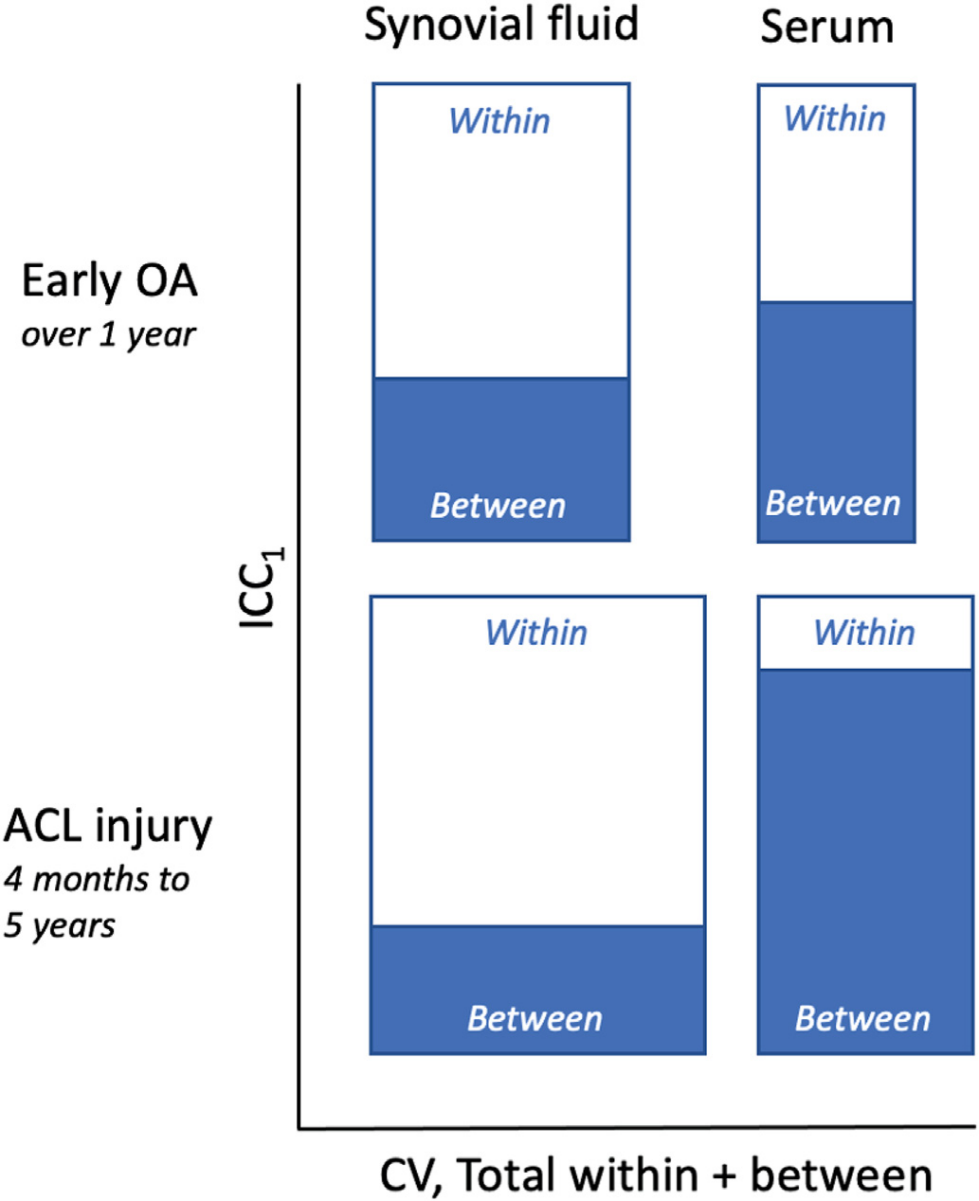
**Bar graph summary of ARGS variability in early-stage OA and after ACL injury.** Bar height represents intraclass correlation (ICC_1_) and the proportion of the variability in ARGS between and within individuals. Bar width is proportional to the sum of the coefficients of variation (CV) between and within individuals. (Based on data in Table 1.)

Biological variation may be regarded as the random variation around a homeostatic set point [37]. The variation around this set point in the individual is the within-subject variation. The homeostatic set point may vary between individuals, which gives the between-subject variation. Our results clearly show that trauma greatly alters the aggrecanase activity, and that joint trauma not only alters the homeostatic set point for the aggrecanase activity, but also increases the variation around the homeostatic set point. Similar to the arguments above on correlation between ARGS in synovial fluid and serum, the alteration after trauma in and around the homeostatic set point appears to differ between synovial fluid and serum. In a graphical summary comparing ARGS variability both between diagnostic groups and between body fluids (Fig. 5), the most striking difference is how the balance between within-individual and between-individual variation after ACL injury were opposite in synovial fluid compared to serum. In synovial fluid the majority of the total variation could be attributed to variation within the individuals over time, whereas in serum it was the variation between individuals that contributed the most. In the OA cohort, the balance between within- and between-individual variation was more even between the body fluids.

This study has some limitations. When studying biological variation in a biomarker, a contributing factor is the analytical source of variation. The assay used to quantify ARGS involves a pre-treatment step that increases the sensitivity of the assay, but also adds handling that may increase the analytical variation. To minimize the effect of analytical variation we analyzed all sequentially taken samples in duplicate on the same analytical run on the same plate. Since only between 6 and 8 subjects were run on each plate, and the between-plate variation is higher than the within-plate variation, the influence of analytical variation is greater on the between-individual variation than on the within-subject variation. We cannot rule out that this may have overall increased the absolute values for the ARGS variability between subjects, but differences in the variability between groups or between fluids remain unaffected. Collection of synovial fluid through knee puncture is more complex and difficult than collection of blood samples, and the resulting volume is often small. This has limited the numbers of subjects with matched samples of synovial fluid and serum in both cohorts. The lack of matched samples at baseline in the ACL injury cohort is a further limitation in this study.

## 5 Conclusion

The biological variation of ARGS is larger in synovial fluid than in serum, and larger after injury than in early-stage OA. Although ARGS concentrations in synovial fluid and serum are correlated, the individual variation is substantial, and a measurement within a patient in one fluid cannot substitute for measurement in the other.

## Contribution

Staffan Larsson acquired and had access to all data, drafted the manuscript and takes responsibility for the integrity of the data and the accuracy of the data analysis. All authors were involved in study conception and design of the study, in the analysis and interpretation of data, and in the critical revision and approval of the final manuscript.

## Role of funding sources

This study received funding from the 10.13039/501100004359Swedish Research Council (VR), Governmental funding of clinical research within the National Health Services (ALF), the 10.13039/501100007949Swedish Rheumatism Association, Greta and Johan Kocks Foundations, and from Galapagos NV in Mechelen, Belgium. Funding sources had no role in the design, collection, and interpretation of the data or the decision to submit for publication.

## Declaration of competing interest

Staffan Larsson has no conflict of interest. André Struglics has no conflict of interest. Stefan Lohmander has no conflict of interest in relation to the subject of this report.
