# Supplementary material for: Biological variation of human aggrecan ARGS neoepitope in synovial fluid and serum in early-stage knee osteoarthritis and after knee injury
Source: Osteoarthr Cartil Open. 2022 Aug 27;4(4):100307. doi: 10.1016/j.ocarto.2022.100307 (PMC9718341; doi:10.1016/j.ocarto.2022.100307)
Supplement: Multimedia component 1 [file mmc1.pdf]

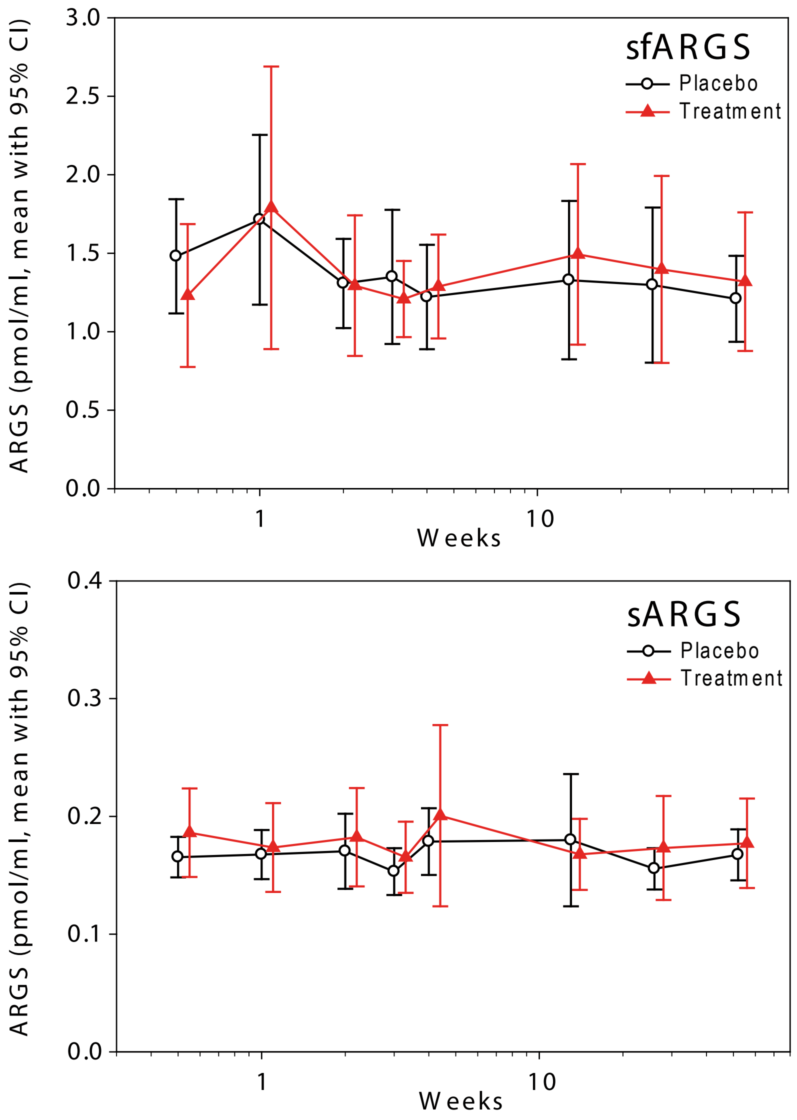

Figure S1. Mean and 95% confidence intervals of ARGS concentrations as treated in the early-stage OA cohort in synovial fluid (sf) and serum (s).

Treatment: intraarticular injection of Hyaluronan, n = 7.

Placebo: intraarticular injection of vehicle, n = 9.
