# Supplementary material for: Biological variation of human aggrecan ARGS neoepitope in synovial fluid and serum in early-stage knee osteoarthritis and after knee injury
Source: Osteoarthr Cartil Open. 2022 Aug 27;4(4):100307. doi: 10.1016/j.ocarto.2022.100307 (PMC9718341; doi:10.1016/j.ocarto.2022.100307)
Supplement: Multimedia component 2 [file mmc2.docx]

| **Supplemental Table S1. Characteristics of the ACL injury cohort** | | | |
| --- | --- | --- | --- |
| Included subjects, n | 120 |  |  |
| Mean age,  years (SD) | 26.1 (4.9) |  |  |
| Female sex,  no. (%) | 32 (27) |  |  |
| Mean body mass index,  kg/m^2^ (SD) | 24.1 (2.9) |  |  |
| Randomization to ACL reconstruction,  Early (Optional delayed) | 61 (59) |  |  |
| Availability of biofluids/ARGS data | Synovial fluid,  no. (%) | Serum, no. (%) | Matched synovial fluid and serum, no. (%) |
| Baseline | 47 (39) | 120 (100) | 47 (39) |
| 4 months | 50 (42) | 64 (53) | 50 (42) |
| 8 months | 48 (40) | 63 (53) | 48 (40) |
| 1 year | 49 (41) | 63 (53) | 48 (40) |
| 2 years | 85 (71) | 119 (99) | 85 (71) |
| 5 years | 68 (57) | 116 (97) | 67 (56) |
