# Supplementary material for: Biological variation of human aggrecan ARGS neoepitope in synovial fluid and serum in early-stage knee osteoarthritis and after knee injury
Source: Osteoarthr Cartil Open. 2022 Aug 27;4(4):100307. doi: 10.1016/j.ocarto.2022.100307 (PMC9718341; doi:10.1016/j.ocarto.2022.100307)
Supplement: Multimedia component 3 [file mmc3.docx]

| **Supplemental Table S2. Early-stage OA ARGS concentrations (pmol/ml) at visits 1 to 8 (baseline to week 52)** | | | | | |  |
| --- | --- | --- | --- | --- | --- | --- |
| **Synovial fluid** |  |  |  |  |  | |
| Visit | Mean | SD | Lower bound 95% CI | Upper bound 95% CI | Between visits differences in means* | |
| BL | 1.371 | 0.483 | 1.114 | 1.628 | p = 0.09 | |
| w1 | 1.746 | 0.803 | 1.319 | 2.174 |  | |
| w2 | 1.301 | 0.408 | 1.084 | 1.519 |  | |
| w3 | 1.287 | 0.444 | 1.051 | 1.524 |  | |
| w4 | 1.250 | 0.390 | 1.043 | 1.458 |  | |
| w13 | 1.400 | 0.625 | 1.067 | 1.733 |  | |
| w26 | 1.340 | 0.624 | 1.008 | 1.673 |  | |
| w52 | 1.257 | 0.402 | 1.043 | 1.472 |  | |
| **Serum** |  |  |  |  |  | |
| Visit | Mean | SD | Lower bound 95% CI | Upper bound 95% CI | Between visits differences in means* | |
| BL | 0.174 | 0.032 | 0.157 | 0.192 | p = 0.22 | |
| w1 | 0.170 | 0.033 | 0.153 | 0.188 |  | |
| w2 | 0.176 | 0.042 | 0.153 | 0.198 |  | |
| w3 | 0.158 | 0.029 | 0.143 | 0.174 |  | |
| w4 | 0.188 | 0.060 | 0.156 | 0.220 |  | |
| w13 | 0.174 | 0.058 | 0.144 | 0.205 |  | |
| w26 | 0.163 | 0.036 | 0.144 | 0.182 |  | |
| w52 | 0.172 | 0.033 | 0.154 | 0.189 |  | |
| * Using an ANOVA with repeated measures with a Greenhouse-Geisser correction. | | | | | | |
|  |  |  |  |  |  | |
