# Supplementary material for: Biological variation of human aggrecan ARGS neoepitope in synovial fluid and serum in early-stage knee osteoarthritis and after knee injury
Source: Osteoarthr Cartil Open. 2022 Aug 27;4(4):100307. doi: 10.1016/j.ocarto.2022.100307 (PMC9718341; doi:10.1016/j.ocarto.2022.100307)
Supplement: Multimedia component 4 [file mmc4.docx]

| **Supplemental Table S3. Pearson correlation and the ratio between sfARGS and sARGS in subjects with early-stage OA and with ACL injury** | | | | |  |
| --- | --- | --- | --- | --- | --- |
| **Early-stage OA** |  |  |  |  | |
| Visit | n | Mean ratio (95% CI) | SD of ratio | Correlation coefficient (95% CI) | |
| BL | 16 | 8.1 (6.3, 9.9) | 3.4 | 0.12 (-0.4, 0.58) | |
| w1 | 16 | 10.4 (7.9, 12.9) | 4.7 | 0.22 (-0.31, 0.65) | |
| w2 | 16 | 7.7 (6.2, 9.3) | 2.9 | 0.12 (-0.4, 0.58) | |
| w3 | 16 | 8.4 (6.6, 10.2) | 3.4 | 0.05 (-0.46, 0.53) | |
| w4 | 16 | 7.1 (5.8, 8.3) | 2.4 | 0.17 (-0.36, 0.61) | |
| w13 | 16 | 8.4 (6.0, 10.9) | 4.5 | 0.28 (-0.25, 0.68) | |
| w26 | 16 | 8.3 (6.1, 10.5) | 4.2 | 0.35 (-0.17, 0.72) | |
| w52 | 16 | 7.5 (5.9, 9.1) | 3.0 | 0.23 (-0.3, 0.65) | |
| *All visits* | 128 | 8.2 (7.6, 8.8) | 3.7 | - | |
| **ACL injury** |  |  |  |  | |
| Visit | n | Mean ratio (95% CI) | SD of ratio | Correlation coefficient (95% CI) | |
| BL | 47 | 90.8 (52.4, 129.2) | 130.7 | -0.03 (-0.32, 0.26) | |
| w16 | 50 | 11.4 (9.5, 13.3) | 6.7 | 0.06 (-0.22, 0.33) | |
| w30 | 48 | 10.6 (8.7, 12.6) | 6.8 | 0.29 (0.01, 0.53) | |
| w52 | 48 | 8.9 (7.6, 10.2) | 4.5 | 0.37 (0.09, 0.59) | |
| w104 | 85 | 9.8 (8.5, 11.2) | 6.3 | 0.13 (-0.08, 0.34) | |
| w260 | 67 | 11.6 (9.1, 14.0) | 10.1 | 0.01 (-0.23, 0.25) | |
|  |  |  |  |  | |
